# Association of psychological variants with functional outcomes among people with spinal cord injury

**DOI:** 10.1038/s41598-021-98808-w

**Published:** 2021-10-13

**Authors:** Mohammed Qasheesh, Mohammad Abu Shaphe, Amir Iqbal, Ahmad H. Alghadir

**Affiliations:** 1 Department of Physical Therapy, College of Applied Medical Sciences, Jazan University, Jazan, Saudi Arabia; 2 Rehabilitation Research Chair, College of Applied Medical Sciences, King Saud University, P.O. Box. 10219, Riyadh, 11433 Saudi Arabia

**Keywords:** Neuroscience, Health care, Medical research, Neurology

## Abstract

This study aimed to investigate the association of psychological variants, including fear, anxiety, and depression, with functional outcomes, including measures of functions and physical performance, among people with spinal cord injury (SCI). An observational study was conducted at the university hospital in Riyadh, Saudi Arabia. Thirty patients, including 19 males (63.33%) and 11 females (36.67%) with a history of traumatic SCI, aged 18–30 years, 1–3 years postinjury T2 and below, with motor complete paraplegia, permanent neurological loss, and the ability to rise from sitting and stand for at least one minute, were included in this study. The Beck Depression Inventory (BDI), State-Trait Anxiety Inventory (STAI), and Fear Avoidance Belief Questionnaire-Physical Activity (FABQ-PA) were used to assess the psychological variants of participants. The Spinal Cord Independence Measure (SCIM) and Walking Index of Spinal Cord Injury (WISCI-II) were used to assess the functional outcomes. Psychological variants showed a strong negative correlation with functional outcomes (*p* < 0.05) among people with SCI. In addition, a significant difference was found between females and males with SCI for the scores of psychological variants and functional outcomes. Psychological variants, including fear, anxiety, and depression, were found to be strongly and negatively associated with functional outcomes, including measures of functions and physical performance, and were especially higher among females than males with SCI. Thus, a higher level of fear, anxiety, and depression results in a lower level of measures of functions and physical performance among people with SCI. Depression is the strongest factor that affects the functional outcomes most among people with SCI.

## Introduction

Spinal cord injury (SCI) disturbs the transmission of signals in the sensory-motor center at the lesion site^1^. Although the incidence of SCI is low, the enormous cost of disability demands remarkable changes in the lifestyle of affected individuals^2,3^. The incidence of SCI in the United States has been estimated to be approximately 12,000 cases per year, whereas in India, the incidence is 10,000 cases per year^2^. Motor vehicle accidents are the most common cause (50.4%), followed by falls (23.8%), violence (11.2%), and sports injuries (9%)^4^. Most SCIs (approximately 80%) occur in males, with the majority of the population (56%) experiencing injury between 16 and 30 years of age^2,4^.

Evidence suggests that psychological variables (i.e., depression, fear, and anxiety) may be correlated with physical functioning among individuals with SCI. As such, they may have a greater impact on physical function when interacting with one another^5^. The National Institute of Mental Health (NIMH) has categorized depression as a mood disorder characterized by the presence of persistent sadness, hopelessness/worthlessness, helplessness, pessimism, and irritability. Individuals with SCI exhibit distinguishable and higher levels of depression and anxiety than the general population; however, it is not a certain reaction to the injury^6^. Of all psychological variables, depression is the most frequently studied among individuals with SCI^7^. Individuals with SCI exhibit signs of depressive disorder, including psychomotor disturbance, changes in appetite, and sleep disturbance^7^. During inpatient rehabilitation, the rates of depression and depressive symptoms have been estimated to range from 20 to 43% and 15% to 50–60%, respectively^8^. Those with SCI who exhibit more severe depressive symptoms tend to experience prolonged lengths of hospitalization, which further results in lower levels of mobility and functional independence at discharge than those free from these symptoms^7^. In addition, these symptoms slow the rate of functional improvement and increase the rate of mortality and morbidity. Furthermore, a prolonged length of hospital stay and rehabilitation program(s) leads to an economic burden on those affected by SCI, their families, and the community^6^.

A previous study reported that the rate of anxiety among individuals with SCI ranges from 10 to 60%, with maximum rates noted immediately before initial discharge^9^. Anxiety resembles fear in an aggravated state of arousal in response to emotional reactions; however, the center of danger remains unclear^10^. Anxiety may encourage extreme preventive behaviors, such as avoiding situation(s) in which an individual anticipates a situation that could potentially be more anxiety-producing or threatening^11^. Anxiety may be a substitute for reported pain; thus, it affects the perception of function(s). In addition, the long-term persistence of these behaviors may lead to more disuse and disability, which in turn decreases opportunities to alleviate the fear of movement and pain. Felt emotions in these patients are of significant interest because the proportion of the body’s sensory mechanism disconnected from the brain varies among these individuals^12^.

Rehabilitation professionals assign a higher priority to outcomes, such as depression, anxiety, fear, and functional performance, while managing individuals with SCI^13^. Decreases in physical capacity among those with SCI are an important loss because they increase risks for complications and are correlated with reduced levels of physical functioning and quality of life. A previous study reported that individuals with SCI may have a lower risk of negative psychological states by receiving effective treatment in the form of cognitive behavioral therapy during rehabilitation^14^. Many studies have identified adaptive patterns for physical functioning and chronic pain^2^; moreover, several experienced clinicians have reported a wide range of adjustments and accommodations in response to psychological behavior(s) and functional limitations^2^.

Accordingly, the current study aimed to investigate the interactions between fear, anxiety, and depression and measures of function and physical performance among individuals who sustained an SCI.

## Methods

### Study design

The present investigation used a cross-sectional observational design. Thirty participants were recruited using a convenience sampling method.

### Ethical considerations

The study obtained ethical approval from the ethics subcommittee at King Saud University, Saudi Arabia. The committee confirmed that the study preserved the human rights, was monitored through the conduct of appropriate research ethics, and was conducted in accordance with the Declaration of Helsinki. All participants returned a completed informed consent form prior to participation in the study.

### Setting

The study was conducted at the outpatient department and rehabilitation unit of our university hospital. A duration of 6 months (from September 2, 2019 to February 3, 2020) was required to complete the study. The vital conditions of the participants were completely stable and would have demonstrated similar stability in their conditions if measurements were repeated at different time points. Demographic characteristics, including age, gender, marital status, educational level, socioeconomic standard, living condition, and social status, were documented prior to the screening for the study. A total of 30 participants with SCI were recruited for this cross-sectional study. Participants were screened and recruited in the study once they satisfied the inclusion criteria. Participants who either did not satisfy the inclusion criteria or met the exclusion criteria of the study were excluded. The inclusion criteria were as follows: participants with a history of traumatic complete SCI; 18 to 30 years of age; chronic SCI, between 1 and 3 years post-SCI; motor complete paraplegia (AIS A); level of injury (T2 and below); permanent neurological loss; and ability to rise from sitting (using upper extremity support) and stand for at least 1 min. Exclusion criteria were as follows: individuals with a history of pathological SCI, associated head injury, dementia and delirium, other neurological disorders, and those who refused to participate.

### Variables

The dependent variables (outcome measures), including psychological variants, such as fear, anxiety, and depression, and independent variables, including measures of functions and physical performance, were measured with their respective assessment tools. Fear, anxiety, and depression were assessed by self-report measures, including the Fear Avoidance Belief Questionnaire-Physical Activity (FABQ-PA), State-Trait Anxiety Inventory (STAI), and Beck Depression Inventory (BDI), respectively. The Spinal Cord Independence Measure (SCIM) and Walking Index of Spinal Cord Injury (WISCI-II) were used to assess the measures of functions (self-care, respiration, and sphincter management and mobility) and physical performance, respectively.

#### Depression

The BDI was used to evaluate the level of depression. Each subject was administered a structured questionnaire after a full explanation of the tool. The questionnaire consisted of 21 questions addressing the severity of the patients’ depression. Patients were required to choose one statement from each group that best described their feelings during the previous 2 weeks, including the day of assessment. Clinical diagnosis of depression was based on criteria from the *Diagnostic and Statistical Manual of Mental Disorders, Fourth Edition* to identify those with elevated levels of depression on the BDI scale. Scores ranging from 0 to 13, 14 to 19, 20 to 28, and 29 to 63 were considered to reflect minimal, mild, moderate, and severe depression, respectively. For the BDI, internal consistency was supported by a mean alpha coefficient of 0.86 and 0.81 among psychiatric and nonpsychiatric patients, respectively^15^.

#### Anxiety

The STAI was applied to evaluate anxiety^16^. This is a self-administered questionnaire consisting of 20 questions addressing the severity of anxiety in an individual. It is theorized that this is a transient emotional state regarded to be a subjective, consciously perceived feeling of apprehension, tension, and aggravated activity of the autonomic nervous system. The subjects were educated about the questionnaire and instructed to opt for the degree to which certain statements pertained to their current feeling(s) of anxiety. The STAI score ranged from lowest to highest (20–80), and the lowest score and highest score indicated lowest and greatest anxiety, respectively. Clinically, an optimal cutoff, ranging from 39 to 40, indicated significant symptoms of state of anxiety. A score between 44 and 50 on the STAI scale identifies a chronically ill patient with an anxiety disorder.

#### Fear-avoidance belief questionnaire

The FABQ-PA was used to measure individuals’ beliefs regarding adverse events related to physical activities^17^. A modified version was adapted to measure fear-avoidance beliefs among individuals with SCI. In the present study, physical activities, such as “standing,” “walking,” “stepping,” and “stand to sit,” were used instead of “bending,” “lifting,” “walking,” and “driving” in the lower-back section of the FABQ-PA. The FABQ-PA computes the level of fear related to physical activities, including interrogating beliefs regarding the relationship of pain with physical activity, and contains 4 items. A 7-point scale was used to rate each item, starting from zero (0) to six (6), with a maximum total score of 24 for all 4 items. The end range anchor “0” represents “completely disagree,” whereas 6 represents “strongly agree.” The middle anchor term is “unsure”. As the therapist explained the questionnaire, the patients rated their fear, avoidance attitude, and beliefs regarding physical activities. Elevated scores indicated a greater level of fear-avoidance beliefs. Scores for the FABQ–PA ranged from 0–24, and scores > 15 were considered to be elevated.

#### Spinal cord independence measure

The SCIM was principally developed to evaluate the performance of activities of daily living and to assess physical function among individuals with SCI. The SCIM assesses three principle areas of function in individuals with SCI: self-care, respiration, and sphincter management and mobility. The items are weighted in terms of their assumed clinical relevance. Self-care involves tasks, such as feeding, bathing, dressing, and grooming, with scores ranging between 0 and 20. Respiration and sphincter management involves respiration, bladder and bowel management, and use of the toilet, with scores ranging from 0 to 40. Mobility addresses task performance in a room and toilet and tasks performed in other areas of the house (indoors) and outdoors, with scores ranging from 0 to 40.

The SCIM was managed by observation. Subjects were asked to perform at their best level. Itemwise scoring of the SCIM was based on observation of tasks performed by the patients who matched the score most suitable to their performance listed in the SCIM evaluation sheet. However, sphincter management, which is not observable, was scored in consultation with the patient’s record or a professional team member who had observed the patient while performing the task. The total score ranged from 0 to 100, and it attributed higher scores when the patient accomplished a task with less support, aids, or medical assistance/compromise than other patients.

#### Walking index of spinal cord injury

Physical performance was measured using the WISCI-II, which assesses a patient’s walking ability in OPD physiotherapy, starting as walking with assistance from bars until independent walking for 10 m. In the walking test session, subjects were asked to ambulate across a 10-m walkway at their preferred speed. Subjects used assistive devices, such as crutches, braces, or walkers, and had minimum to maximum assistance according to their ability and the severity of their impairment. The test was terminated when the subjects felt too tired to continue. The assessment index includes an order of ranking based on the severity of impairment, starting from most severe (0) to least severe (20), depending on the use of braces, devices, and physical support of ≥ 1 person. This order of levels indicates that each successive level is less impaired than the previous level. According to this scoring, the therapist judged and assigned a level at which the patient was considered to be safe.

### Statistical analysis

Data were analyzed using SPSS version 22.0 (IBM Corporation, Armonk, NY, USA). The independent *t* test was used to compare means of psychological variables, measures of function, and physical performance between males and females. The Pearson product moment correlation coefficient (PPMCC) was used to compare normally distributed continuous variables to assess the association among psychological variables, measures of function, and physical performance. A series of linear regression analyses were performed to assess relationships among psychological variables and their interaction effects on measures of function and physical performance. Differences with p ≤ 0.05 were considered to be statistically significant.

### Ethical approval and consent to participate

The study received ethical approval from the ethics subcommittee at King Saud University, Saudi Arabia. All participants were adults aged more than 18 years and returned a completed informed consent form prior to participation in the study.

## Results

Forty-nine participants (31 males, 18 females) with SCI were potentially eligible for this study. Eleven participants did not match the eligibility criteria, 5 declined to participate due to unavailability of time, and 3 did not have any reason. Thirty (19 males, 11 females) participants with a response rate of 69.77% met the eligibility criteria and were successfully included in the study. Data are expressed as the mean and standard deviation (SD), as indicated. The mean ages of the females and males in the present study were 27.5 ± 5.3 years and 25.3 ± 4.4 years, respectively. Demographic characteristics of the participants are explained as an average age of 18–30 years (females = 27.5 ± 5.3 and males = 25.3 ± 4.4, years), gender (males = 19 (63.33%) and females = 11 (36.67%)), marital status (single = 14, married = 12, divorcee = 4), educational level (undergraduate = 3, graduate = 11, postgraduate = 16), socioeconomic status (lower = 6, middle = 20, upper = 4), living condition (independently = 7, with family = 23), and social support (yes = 29, no = 1) for this study. Data representing psychological variants (fear, anxiety, and depression), measures of function, and physical performance of the study population were analyzed as follows.

### Comparison of variables between males and females

The results of the independent *t* test indicated that females with SCI perceived significantly (*p* < 0.05) greater fear, anxiety, and depression and exhibited lower measures of function and physical performance than males with SCI (Table 1).

**Table 1:** Comparison of psychological variants, functional outcomes between males and females with SCI.

Variables	Gender		Independent t-test		p-value
	Males	Females	t-value	Df	
BDI	26.06 ± 7.94	35.22 ± 10.08	2.3558	10.6	0.038*
STAI-S	38.25 ± 6.55	44.88 ± 8.57	2.14	10.9	0.05*
FABQ-PA	8.83 ± 5.63	14.88 ± 7.28	2.29	10.9	0.04*
SCIM	65.51 ± 6.33	58 ± 7.43	− 2.75	11.6	0.017*
WISCI	9.12 ± 3.68	5.66 ± 3.42	− 2.62	13.9	0.02*

*Significant at p ≤ 0.05; WISCI-II: Walking Index of Spinal Cord Injury (WISCI-II); SCIM: Spinal Cord Independence Measure; FABQ-PA: Fear-avoidance belief questionnaire; STAI-S: State trait anxiety inventory; BDI: Beck Depression Inventory.

### Psychological variants (fear, anxiety, and depression)

The mean BDI scores for males and females were 26.06 ± 7.94 and 35.22 ± 10.08, respectively. The mean STAI-S scores for males and females were 38.25 ± 6.55 and 44.88 ± 8.57, respectively. The mean FABQ-PA scores for males and females were 8.83 ± 5.63 and 14.88 ± 7.28, respectively (Table 1).

### Measures of function (SCIM) and physical performance (WISCI-II)

The mean SCIM scores for males and females were 65.51 ± 6.33 and 58 ± 7.43, respectively. The mean WISCI-II scores for males and females were 9.12 ± 3.68 and 5.66 ± 3.42, respectively (Table 1).

### Association among variables (bivariate correlation)

PPMCC analysis revealed a significant negative correlation between each psychological variable and all measures of function and physical performance in individuals with SCI (Table 2). Compared with males, females demonstrated a significantly (*p* < 0.05) higher correlation between each psychological variable and measures of function and physical performance (Table 3).

**Table 2:** Bivariate correlation between psychological variants and functional outcomes among people with SCI.

Variables	SCIM		WISCI	
	r value	*p* value	r value	*p* value
BDI	− 0.97*	0.0001*	− 0.94*	0.0001*
STAI-S	− 0.93*	0.0001*	− 0.91*	0.0001*
FABQ-PA	− 0.97*	0.0001*	− 0.91*	0.0001*

*Significant at p ≤ 0.05; WISCI-II: Walking Index of Spinal Cord Injury (WISCI-II); SCIM: Spinal Cord Independence Measure; FABQ-PA: Fear-avoidance belief questionnaire; STAI-S: State trait anxiety inventory; BDI: Beck Depression Inventory.

**Table 3:** Bivariate correlation between psychological variants and functional outcomes among genders.

Variables	SCIM		WISCI	
	r value	*p* value	r value	*p* value
**BDI**				
Male	− 0.96*	0.0001*	− 0.95*	0.001*
Female	− 0.99*	0.0001*	− 0.95*	0.001*
**STAI-S**				
Male	− 0.89*	0.0001*	− 0.90*	0.0001*
Female	− 0.99*	0.0001*	− 0.93*	0.0001*
**FABQ-PA**				
Male	− 0.96*	0.0001*	− 0.89*	0.0001*
Female	− 0.98*	0.0001*	− 0.94*	0.0001*

*Significant at p ≤ 0.05; WISCI-II: Walking Index of Spinal Cord Injury (WISCI-II); SCIM: Spinal Cord Independence Measure; FABQ-PA: Fear-avoidance belief questionnaire; STAI-S: State trait anxiety inventory; BDI: Beck Depression Inventory.

### Regression model (linear regression)

Regression modeling revealed that only depression demonstrated a significant association with both the measures of function (Y = − 0.329; p = 0.001) and physical performance (Y = − 0.319; p = 0.001), whereas fear demonstrated a significant association only with measures of function (Y = − 0.591; p = 0.0001). However, there was no significant (p > 0.05) association between anxiety and measures of function (Y = − 0.043; p = 0.615) or physical performance (Y = − 0.052; p = 0.001) in those with SCI (Table 4).

**Table 4:** Regression model.

Variables		Linear regression	
Independent	Dependent	Y-value	p-value
SCIM	BDI	− 0.329**	0.001**
	STAI-S	− 0.043^ns^	0.615^ns^
	FABQ-PA	− 0.591**	0.0001**
WISCI	BDI	− 0.319**	0.001**
	STAI-S	− 0.052^ns^	0.56^ns^
	FABQ-PA	− 0.049^ns^	0.63^ns^

WISCI-II: Walking Index of Spinal Cord Injury (WISCI-II); SCIM: Spinal Cord Independence Measure; FABQ-PA: Fear-avoidance belief questionnaire; STAI-S: State trait anxiety inventory; BDI: Beck Depression Inventory.

NS- Not significant; **Highly significant at *p* < 0.01; Y-Regression.

Depression (according to the BDI) was revealed to be a more strongly associated psychological variable with measures of function and physical performance in the regression model, meaning that depression highly affected the measures of function and physical performance in individuals with SCI (Table 4). In contrast, anxiety did not affect measures of function and physical performance in those with SCI.

## Discussion

The primary findings of the present study supported the proposed hypothesis that psychological variables, including fear, anxiety and depression, are significantly correlated with measures of function and physical performance among individuals with SCI. These variables exhibited a significant inverse correlation with measures of function and physical performance, which suggests that higher levels of fear, anxiety, and depression could lower measures of function and physical performance, as reflected by the bivariate correlation model^18^.

A previous study reported that higher levels of anxiety among individuals with SCI are correlated with a lower level of physical functioning and advised addressing anxiety as a problem in addition to physical function while managing individuals with SCI^19^. Furthermore, a postulated model described the role of anxiety in declining physical function using a cognitive mechanism and concluded that emotional experience can prompt the emergence of symptoms and intended functions or vice versa^20^.

The regression model in our study revealed that anxiety was not significantly correlated with measures of function and physical performance among individuals with SCI. Surprisingly, our bivariate correlation demonstrated a contradictory result, in which anxiety *was* significantly associated with the measures of function and physical performance.

Pain has a significant impact on activity levels and is associated with a reduction in global self-rated health and higher levels of psychological distress^21^, which can predispose patients because of the pain experienced, which can lead to potential amplification of the pain experience^22^. To some extent, psychological variables may account for differences in reported pain that may also affect the perception of function^23^. The proposed behavioral mechanism, in which the emotional aspect of pain elevates the habit to prevent activities related to the pain, enters into a vicious cycle of decreased physical functioning and weakened muscular strength, which in turn results in more disability among individuals with SCI. In accordance with previous findings on low back pain, a significant relationship between higher fear-avoidance beliefs and worse physical function was found in the present study.

Similar results have been reported in a study by Nicholson Perry et al.,^24^ in which the intensity of pain demonstrated a strong correlation with depression, anxiety, and life interference related to pain. Furthermore, pain catastrophizing demonstrated a significant and positive correlation with depression and anxiety scores and a negative correlation with physical function scores.

Another study reported a similar level of pain intensity, in which pain interference activities from different areas were associated with the symptoms of pain and psychological factors^21^. The relationship between frequent interference and pain intensity demonstrates that individuals with SCI experiencing different types of high-intensity pain are more prone to encounter frequent interference from various types of activities of daily living than normal healthy individuals^25^. The relationship between pain and depression develops over time. Reduced pain has a greater effect on reducing depression than decreased depression has on pain^20^.

In addition to the above psychological variables in the present study, anxiety and fear were not significantly associated with the measure of physical performance in the regression model. Because there is a reduced level of activity in the proposed fear-avoidance model, we assumed that there must be a relationship between actual physical tasks and measures of function and physical performance in the study.

Depression was found to be the most important and prevalent factor associated with measures of function and physical performance in the current study. Similar results were reported by Hughes et al.^20^, in which severe physical disability was correlated with the risk of severe depression. Similarly, a previous study reported a negative correlation between functional independence and depression^6^. Interestingly, our study also surpassed the 30% estimation for major depression, which previous studies have described^5^. According to our findings, the rate of major depression was 40%. The high rate of depression in the present study may reflect several factors. This study used the BDI to measure depression because it is the most sensitive measure, with a sensitivity of 83.3% and a specificity of 90.8%, as reported by previous studies reviewed by Kalpakjian et al.^26^.

The continuous frustration occurring with prolonged rehabilitation with limited progress in outcomes, a lack of social skill integration into the community to obtain social support, and result of physical difficulties in the activities of daily living leading to poor quality of life may be strongly correlated with the lack of reduction in depression and anxiety. The assumption that “time heals” may neglect the intricate processes associated with adjustment to the state of SCI^27,28^.

On returning home from institutional rehabilitation, individuals with SCI may experience increased levels of depression due to their increased dependency, lack of social skill, lack of social support other than family, and lack of employment opportunities^29^.

The kingdom of Saudi Arabia has been marking a significant presence among countries, progressing to its fastest demographic, cultural, and economic transition in its population and advancing to a higher standard of living, longer life expectancy, better health care system, and stronger education; simultaneously, also it has been facing challenges, such as a sedentary lifestyle, the pressure of being modernized, a lack of social skill and social support, social isolation, an increase in the number of chronic mental diseases, including depression, pre-existing stigma of mental disorders, scarcity of resources supporting mental health, and direct and indirect rise in costs of treating depression^30–33^. Previously, one study targeted a population (secondary school students aged between 16–20 years) and conducted a gendered comparison of possible risk factors that contribute to depression using the BDI. Furthermore, a prevalence of depressive symptoms 1.5 times higher in girls than in boys was revealed, and sex, birth order, history of psychiatric illness, loss of relatives, and chronic diseases in a family tree were considered the most significant risk factors involved^33^.

Similarly, a previous study reported that psychological factors, such as stress and social isolation, are more potent than disability-related or demographic factors in the development of depression in women than men with SCI^32–35^. In another study, anxiety and depressive symptoms were found to be positively correlated with sex and educational level among individuals with traumatic SCI in Saudi Arabia. In addition, the study also reported that higher education at the university level was moderately associated with higher levels of anxiety and depression in the same population^34^. In contrast to previous thought, this was the first meta-analysis study (2010) that reported a lower prevalence of depression and a low risk of developing depressive symptoms among the population in Saudi Arabia, regardless of gender. Additionally, men were found to be less likely to develop depressive symptoms than women in Saudi Arabia^32^. Therefore, more scientific studies are required to provide insight into the prevalence and risk of developing symptoms of psychological variants in Saudi Arabia to attract the attention of health care practitioners, policy makers, and management systems taking solid steps to lower the psychological variants among individuals with SCI.

The highly significant results of this study could be explained by the excellent reliability and validity of the scales chosen, including the BDI (sensitivity, 83.3%; specificity, 90.8%); STAI-S (high intraclass correlation coefficient [ICC, 0.39 to 0.89]); FABQ-PA (ICC, 0.66 and internal consistency 0.79); SCIM (correlation coefficient rate, 0.66–0–98); and WISCI (intrarater reliability, 0.97).

The current study, however, had some limitations. First, the small sample size restricted our ability to generalize the findings. The present study addressed measures of function and physical performance only. However, except for gender, other factors, such as age, marital status, socioeconomic status, education level, living standard, social support, frequency of prior hospitalization, coping skills, and self-efficacy, were also considered to be important covariates that could affect the psychological and functional measures and were not evaluated for their causal relationship with these variables in this study. The self-report nature of the study restricted our ability to draw causal relationships and may have produced some response biases.

Future research should examine the effects of fear-avoidance beliefs and anxiety on rehabilitation programs targeted at improving physical function. Additionally, the potential use of battery-operated device(s) would better record complete measures of physical performance. In addition, education level, socioeconomic status, social support system, marital status, living condition, coping skills, and self-efficacy are also important behavioral and other factors that should also be addressed for their relationship with psychological variants and measures of function in future investigations.

## Conclusion

The results of the present study suggest that higher scores on psychological variables were significantly and independently associated with poorer physical function according to a bivariate correlation model. In addition, depression was found to be the strongest factor among all the psychological variables and exhibited a strong relationship with measures of physical performance. Thus, clinicians should target psychological variants, including depression, set specialized training, and develop follow-up programs to maximize the potential of physical capacity and physical performance among individuals with SCI.

## Data Availability

The datasets generated and/or analyzed during the current study are available from the corresponding author on reasonable request.
